# Exposure to 3-Nitropropionic Acid Mitochondrial Toxin Induces Tau Pathology in Tangle-Mouse Model and in Wild Type-Mice

**DOI:** 10.3389/fcell.2019.00321

**Published:** 2020-01-14

**Authors:** Inbal Lahiani-Cohen, Olga Touloumi, Roza Lagoudaki, Nikolaos Grigoriadis, Hanna Rosenmann

**Affiliations:** ^1^The Department of Neurology, The Agnes Ginges Center for Human Neurogenetics, Hadassah Hebrew University Medical Center, Jerusalem, Israel; ^2^B’ Department of Neurology, AHEPA University Hospital, Thessaloniki, Greece

**Keywords:** tau protein, neurofibrillary tangles, tauopathy, mitochondria, mitochondrial dysfunction, oxidative stress, 3-nitropropionic acid

## Abstract

Oxidative stress, particularly of mitochondrial origin, plays an important role in the pathogenesis of neurodegenerative disorders, including Alzheimer’s disease (AD) and other tauopathies. Controversies regarding the responses of tau phosphorylation state to various stimuli causing oxidative stress have been reported. Here we investigated the effect of 3-nitropropionic acid (3NP), a mitochondrial toxin which induces oxidative stress, on the tangle-pathology in our previously generated double mutant (E257T/P301S, DM) -Tau-tg mice and in WT-mice. We detected an increase in tangle pathology in the hippocampus and cortex of the DM-Tau-tg mice following exposure of the mice to the toxin, as well as generation of tangles in WT-mice. This increase was accompanied with alterations in the level of the glycogen synthase kinase 3β (GSK3β), the kinase which phosphorylates the tau protein, and in the phosphorylation state of this kinase. A response of microglial cells was noticed. These results point to the involvement of mitochondrial dysfunction in the development of the tangle-pathology and may suggest that interfering with mitochondrial dysfunction may have an anti-tangle therapeutic potential.

## Introduction

Emerging evidence suggests that oxidative stress plays an important role in the pathogenesis of neurodegenerative disorders, including Alzheimer’s disease (AD). Oxidative stress results from the imbalance between the productions of reactive oxygen species (ROS) and antioxidant defenses. Growing evidence has highlighted expanded roles of mitochondria in modulating oxidative stress, with the mitochondria being the major producer of ROS in cells, and the bulk of mitochondrial ROS generated at the electron transport chain (reviewed in (Cui et al., 2012; Chen et al., 2018). ROS react with lipids, proteins, and nucleic acids causing oxidative damage to these macromolecules, thereby inducing tissue damage and cell death, characteristic to the pathophysiology of neurodegenerative disorders, including AD (Smith et al., 1997; Markesbery and Lovell, 1998; Andersen, 2004; Zhu et al., 2004a, b). Most of the known genetic, medical, environmental, and lifestyle-related risk factors for AD are associated with increased oxidative stress, and similarly various protective factors for AD (nutrients and agents, as well as lifestyle-related factors) aimed to attenuate oxidative damage (Zhu et al., 2007; Su et al., 2008).

The tau pathology, its pathologically phosphorylated state and the formation of neurofibrillary tangles (NFTs), are key stages in the pathogenesis of AD. The tangles are considered the brain pathology that best correlates with dementia, better than the amyloid-pathology (Braak and Braak, 1991; Arriagada et al., 1992; Bierer et al., 1995). Therefore, elucidating the regulation of the tau pathology is critical for our understanding of AD pathogenesis. Moreover, in addition to the contribution of the tangles to the pathogenesis of AD which is a secondary tauopathy (tangles develop secondarily to amyloid pathology), tangles are also the characteristic pathology affecting other neurodegenerative dementia diseases, i.e., primary tauopathies [such as frontotemporal dementia with parkinsonism (FTDP), Pick’s disease and progressive supranuclear pulsy (PSP)] where no amyloid pathology is present. Oxidative stress in brain is evident also in these primary tauopathies (Castellani et al., 1995; Hartzler et al., 2002; Muntané et al., 2006). The relation between oxidative stress and tau/tangle pathology is not clear: is oxidative stress an early causal factor in the pathophysiological process or is it rather a consequence of the cell damage induced by tau hyper-phosphorylation. Variable responses - and even contradictive – of tau phosphorylation state to various triggers/stimuli causing oxidative stress in neuronal cell cultures have been reported: oxidative stress induced by acrolein (Gómez-Ramos et al., 2003), hydroxynonenal (Mattson et al., 1997), homocysteine (Ho et al., 2003), mercury (Olivieri et al., 2000), haloperidol (Benítez-King et al., 2010), and mammalian target of rapamycin (mTOR) activator (Taga et al., 2011) – increased the phosphorylation of tau, while oxidative stress induced by hydrogen peroxide (LoPresti and Konat, 2001; Galas et al., 2006), ultraviolet-light (Olivieri et al., 2001), and menadione (Ko et al., 1997) – caused dephosphorylation of tau protein or increased tau turnover (Poppek et al., 2006), and iron induced stress modified the pattern of tau phosphorylation (Egaña et al., 2003) in cell cultures. Responsiveness of tau to oxidative stress has also been reported in animals, with variable results: no increase in tau phosphorylation but only in tau-induced cell cycle activation was reported in a tauopathy drosophila model following genetic inactivation of antioxidant mechanisms (Dias-Santagata et al., 2007).

Suppression of several phenotypes of tauopathy in mice was reported when mild oxidative stress was generated by alloxan (Yoshiike et al., 2012). An opposite effect showing an increase of tau phosphorylation was reported in genetic mouse models for oxidative stress, particularly those associated with mitochondrial dysfunction: lacking the defensive enzyme superoxide dismutase 2 (Melov et al., 2007), or defective in mitochondrial aldehyde dehydrogenase 2 activity (Ohsawa et al., 2008). The interaction between mitochondrial dysfunction and tau pathology was assessed genetically by crossbreeding mutant tau mice expressing tangles with the harlequin mutant mice (which suffer from mitochondrial dysfunction and oxidative stress due to the mitochondrial apoptosis-inducing factor, AIF, depletion), showing age-dependent mutual reinforcement of the tau pathology and mitochondrial dysfunction of complexes I–IV (Kulic et al., 2011).

In the present study we assessed the responsiveness of a tg mouse tangle model to pharmacologically induced mitochondrial dysfunction and oxidative stress, using the 3-nitropropionic acid (3NP) toxin. The prime neurotoxic mechanism of 3NP, a natural environmental toxin obtained from various plants, is due to its irreversible, covalent binding. Subsequently, 3NP inhibits succinate dehydrogenase (SDH), an enzyme of the citric acid cycle that transfers electrons to the electron transport chain via its complex II function (Huang et al., 2006). It has been reported that 3NP produces selective basal ganglia (striatum) and cortex hippocampal lesions and dystonia in humans (Karanian et al., 2006). Accumulating data indicates that 3NP produce free radicals and consequent disturb glutathione redox cycle (Kumar et al., 2006; Mao et al., 2006), and inactivates Glycogen synthase kinase 3β (GSK3β) (Crespo-Biel et al., 2010; El-Abhar et al., 2018). Injection of this toxin in mice is in use for studying neurodegenerative diseases, particularly the striatum in Huntington’s disease (Kodsi and Swerdlow, 1997), but also in AD (Arias et al., 2002; von Arnim et al., 2005, 2006). Tau tg mice subjected to acute neuronal injury using 3NP showed increased interstitial fluid tau and plasma tau, yet no data regarding the effect of 3NP on brain tau pathology (phosphorylated tau and tangles) was reported (Yanamandra et al., 2017).

We injected the 3NP to our previously generated model for tauopathy, which expresses two severe mutations under the regulation of the original tau promoter [double-mutant (DM) tau transgenic (tg) mice] (Rosenmann et al., 2008). We were interested to see whether the toxin can accelerate the tau pathology in the cortex and in the hippocampus, regions which are directly affected in the tg-taupathy-model (expressing mutant human tau); and whether 3NP can also affect the brain of WT-mice, which express only the endogenous non-mutant mouse tau. We detected that exposure to the complex II inhibitor 3NP lead to the exacerbation of tau pathology in the cortex and hippocampus of DM-tau mice, and to the generation *de novo* of tau pathology in WT mice, accompanied with alterations in the GSK3β level and phosphorylation state, and also with a microglial response.

## Materials and Methods

### Animals

We used the (E257T/P301S) human double mutant tau protein (DM-Tau-tg mice) mouse model regulated by the natural-tau-promoter, previously generated and described by us (Rosenmann et al., 2008), further crossed with C57Bl mice for more than 6 generations. Tg offspring were identified by polymerase chain reaction analysis of tail genomic DNA. The non-tg littermates were used as the non-tg mice (WT-mice). The experiments were approved by the Institutional Ethics Committee of The Hebrew University of Jerusalem.

### 3NP Injection Protocols

We used two different protocols of 3NP injection. (1) Moderate long term 3NP treatment of DM-tau-tg mice: 5 month old DM-tau-tg mice (about 1 month before onset of brain tau-pathology in this model) (*n* = 4/group) were injected IP with 3NP (Sigma Aldrich Israel) (15 mg/kg) dissolved in saline or with saline only, two times a week for 5 months. (2) Acute short term 3NP treatment of DM-tau-tg mice and WT-mice: 13 month old DM-tau-tg mice (about 7 months following onset of brain tau-pathology in this model) (*n* = 7/group) and 13 months old WT-mice (*n* = 4/group) were injected IP with 3NP (15 mg/kg) dissolved in saline or with saline only, in every other day for 1 month. [We used a lower concentration of 3NP than that in use in the 3NP injected HD or MSA models (Stefanova et al., 2005; Chakraborty et al., 2014)].

### Neuropathological Examinations

#### Tissue Collection

Animals were sacrificed at the end of each experiment under deep anesthesia and were rapidly transcardially perfused with PBS, followed by 4% paraformaldehyde in PBS (pH 7.2, ice cold). Brains were quickly removed and post-fixed for 20 h in the same fixative and embedded in paraffin. Histological staining and immunohistochemistry were performed on 6 μm serial brain sections.

#### Histology and Immunohistochemistry (IHC)

Paraffin embedded sections were silver-impregnated by the Gallyas-silver method that stains tangles and nerve cell processes fine fibrils containing the abnormal tau protein in AD and tauopathies (Gallyas, 1971). Detection of NFTs was also performed by IHC using the AT8 and AT180 mouse monoclonal Abs (Innogenetics, Ghent, Belgium), which recognize tau phosphorylated at 202/205 and 231, respectively, epitopes characteristic to tau-pathology in AD/tauopathies, as well as in our DM-Tau-tg mice used in this study, as previously described in detail (Rosenmann et al., 2008). Exposure of total Tau protein was accomplished with a rabbit monoclonal to Tau (EPR22524-95, Abcam). Microglial cells were immune-stained with the Iba1 Ab (WAKO, Chemicals United States). GSK-3β was immune-stained with anti-GSK3β (phospho S9, Abcam) [GSK3β (S9)] and anti-GSK3β (phospho Y216, Abcam) [GSK3β (Y216)] rabbit polyclonal Abs, as well as with anti-GSK3β (3D10) mouse monoclonal Ab for total GSK3β. Briefly, paraffin sections were deparaffinized and hydrated in xylene and alcohol solutions, and antigen retrieval was performed with citrate buffer pH 6 or with EDTA pH 8.5 in a steamer device (Braun, Kronbrg/Taunus, Germany) for 60 min. Endogenous peroxidase was blocked with 0.3% H2O2 in methanol and was followed by incubation in the appropriate blocking buffer (10% Fetal Bovine Serum in TBS) for 30 min. Sections were incubated overnight at 4°C with the primary Abs AT8, AT180, IBA 1, GSK3β (S9), GSK3β (Y216) and GSK3β (3D10). Immunostaining was visualized using the Mouse-on-Mouse system (Vector Laboratories, Burlingame, CA, United States), secondary antibody goat ant rabbit (Vector), goat anti-mouse (Vector) and then incubated with Avidin – Peroxidase (Sigma). The 3,3′-diaminobenzidinetetrahydrochloride (DAB) (DAKO) was used as chromogen and sections were counterstained with hematoxylin. Finally, sections were dehydrated in graded ethanol and covered with Entellan.

In order to identify the phenotype of microglia M1/M2-double immunofluorescence was performed as previously described (Lourbopoulos et al., 2011). In order to identify the presence of GSKβ in the neurons (NeuN, Millipore) double immunofluoerescent was performed, for GSKβ (S9)/NeuN. Goat anti-mouse IgG 555 (Biotium, CF 555) and chicken anti-rabbit IgG (Biotium, CF 488A), were used as secondary antibodies. Slides were mounted with Dapi (Biotium).

#### Neuropathological Evaluation

Sections were examined under optical and fluorescent microscope Nikon Eclipse Ci and Zeiss Axioplan-2 with the aid of two CCD cameras (Basler acA1920 and Nikon DS-5M, respectively). The evaluation was performed on the area of the cortex and the hippocampus. In average 30 optical fields were examined per each group under the magnification of 40X. Results were expressed as positive cells/mm^2^ (for Gallyas, AT8, AT180 and total Tau), area/mm^2^ [for GSKβ(S9), GSKβ(Y216) and 3D10]. The microglial activation state was quantified as cells/mm^2^ and by determining the ramification index (RI), ranging from 0 for ramified “resting” cells to1 for “active” ameboid cells (Wilms et al., 1997).

### Data Analysis

Analysis of the data was performed using the GraphPad Prism7.0 software. The normality was tested using the Shapiro–Wilk and Kolmogorov–Smirnov tests. Parametric data were analyzed using unpaired *t*-test. Non-parametric data were analyzed using Mann–Whitney test. All determinations were made with a 95% confidence interval and were considered significant at the *p* < 0.05 level. Values of all scale data are expressed as mean ± SE.

## Results

### Increased Tau-Pathology in DM-Tau tg Mice Injected With 3NP

**FIGURE 1:**
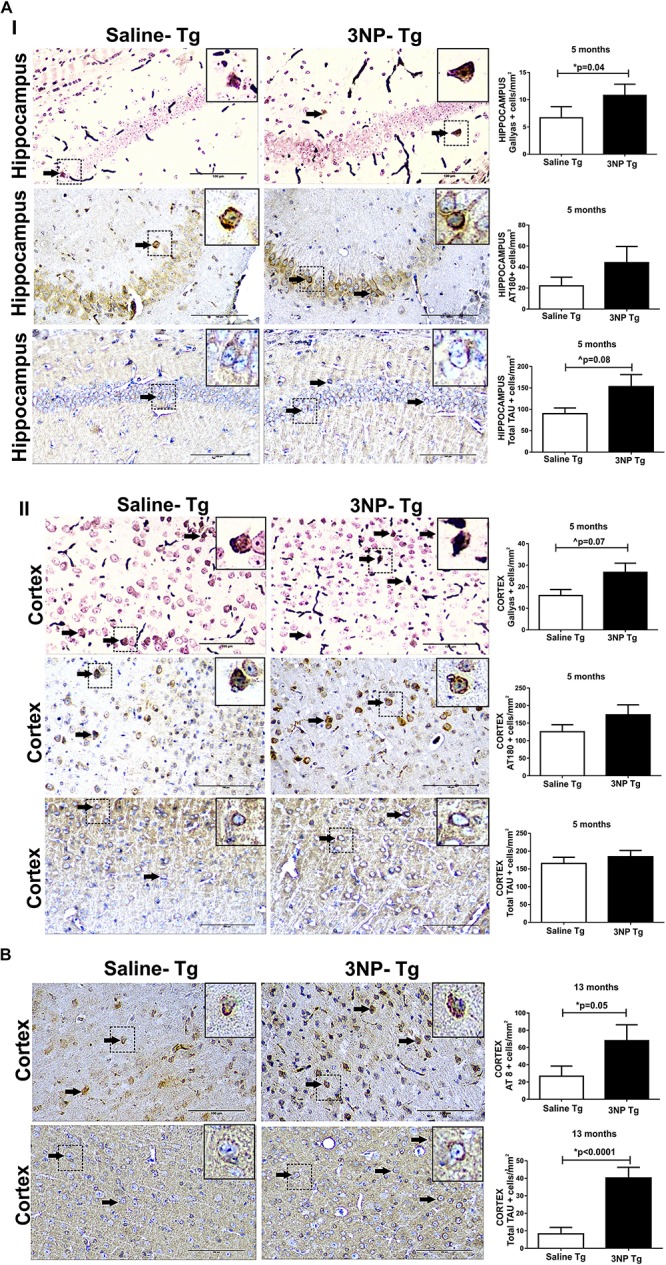
Increased tau pathology in DM-Tau-tg mice exposed to 3NP. **(A)** Increased tau pathology in 5-month old DM-Tau-tg mice treated with a moderate long-term protocol of 3NP exposure (IP injection two times a week for 5 months). Significant increase in burden of Gallyas positive cells (cells/mm^2^) in the hippocampus of 3NP-DM-Tau-tg mice relative to saline- DM-Tau-tg mice (*p* = 0.04), with similar tendency of increase in burden of AT180 positive cells (cells/mm^2^). A trend of higher total-tau (tau-5 positive cells/mm^2^) (*p* = 0.08) in the 3NP-treated mice relative to saline-treated mice **(I)**. Increase in burden of Gallyas positive cells in the cortex of 3NP-DM-Tau-tg mice relative to saline- DM-Tau-tg mice (trend p = 0.07), with similar tendency of increase in burden of AT180 positive cells. No significant difference in the total-tau in the 3NP-treated mice relative to saline-treated mice **(II)**. **(B)** Increased tau pathology in 13-month old DM-Tau-tg mice treated with an acute short-term protocol of 3NP exposure (IP injection every other day for 1 month). Increase in burden of AT8 positive cells in the cortex of 3NP-DM-Tau-tg mice relative to saline- DM-Tau-tg mice (*p* = 0.05). Higher level of total-tau (tau-5 positive cells/mm^2^) (*p* < 0.0001) in the 3NP-treated mice relative to saline-treated mice. Negative control staining for Gallyas, AT8 and AT180 is presented in Supplementary Figure 1. Arrows show positive stained cells. Scale bars = 100 μm. Magnification of the photos in X40, dashed line indicate positive cells from each photo transferred in higher magnification insert photos.

Using the moderate long term protocol of 3NP injection (IP injection two times a week for 5 months) in 5-month old DM-tau tg mice (3NP -DM-Tau-tg mice, about 1 month before onset of brain tau-pathology in this model) showed a significant increase of tau pathology in the hippocampus, as indicated by the increase of 61.5% in the Gallyas-positive cell staining for NFTs relative to saline treated mice (*p* = 0.04), with a similar tendency (*p* = 0.17) of increase in phosphorylated tau burden, indicated by the 100% increase in the burden of AT180 cells/mm2 (Figure 1AI). A similar result pointing to an increase in tau pathology in 3NP -DM-Tau-tg mice under this protocol of 3NP exposure was also noticed in the cortex: increase of 67.8% Gallyas-positive cells/mm^2^ relative to the saline treated mice (trend, *p* = 0.07), with such a tendency of increase of 38.5% of AT180 positive cells cells/mm^2^ (*p* = 0.18) (Figure 1AII). An indication that the increase in the pathological/phosphorylated tau in the brain of tg-mice exposed to 3NP was accompanied with an increase of the total tau was noticed by the increase of 70.3% of tau5 cells/mm^2^ (trend, *p* = 0.08) in the hippocampus, yet not in the cortex (Figure 1A). An increase in tau pathology was also detected when using the acute short term protocol of 3NP treatment (injection every other day for 1 month) in 13-month old DM-tau tg mice (an age in which NFT deposition is already established in this model), as detected particularly by the increase of 153.8% in the AT8 staining for phosphorylated tau in the cortex (*p* = 0.05) relative to saline treated mice, accompanied with an increase of 389.2% in the total-tau (*p* < 0.0001) (Figure 1B). Such differences were not detected in the hippocampus (data not shown).

### Evidence for Tau-Pathology in WT-Mice Injected With 3NP

**FIGURE 2:**
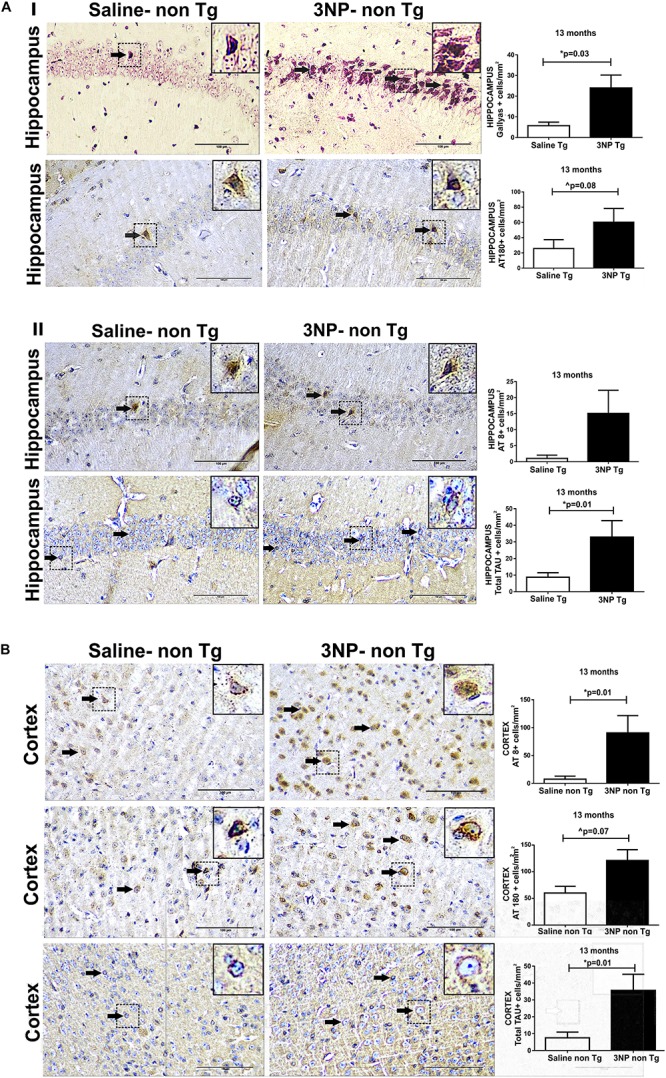
*De novo* generation of tau pathology in WT-mice exposed to 3NP. Significantly more tau pathology in the hippocampus **(A)** of 13 -month old 3NP-WT- mice under the acute short-term protocol of 3NP exposure than in the saline-DM-Tau-tg mice: more Gallyas positive cells (*p* = 0.03) and AT180 positive cells (trend 0.08) **(I)**, with similar tendency in AT8 positive cells (cells/mm^2^). Higher level of total-tau (tau-5 positive cells/mm^2^) (*p* = 0.01) in the 3NP-treated mice relative to saline-treated mice **(II)**. Also significantly more tau pathology detected in the cortex **(B)** of these 3NP-WT- mice relative to saline-WT- mice: more AT8 positive cells (0.01), and AT180 positive cells (trend *p* = 0.07) Higher level of total-tau (tau-5 positive cells/mm^2^) (*p* = 0.01) in the 3NP-treated mice relative to saline-treated mice. Arrows show positive stained cells. Scale bars = 100 μm. Magnification of the photos in X40, dashed line indicate positive cells from each photo transferred in higher magnification insert photos.

In order to see whether 3NP can not only increase tangle pathology in genetically engineered mice which generate NTFs, but can also lead to *de novo* formation of tau pathology originating from non-mutant tau in WT-mice, we exposed also 13 months old WT-mice to 3NP for 1 month. High burden of cells positive for Gallyas staining were detected in the hippocampus of 3NP injected mice (3NP-WT-mice), which was 320.1% higher than the limited burden detected in the saline injected mice (*p* = 0.03). Also an increase of 134.6% in the burden of phosphorylated tau AT180 positive cells/mm^2^ (trend, *p* = 0.08) and a similar tendency (*p* = 0.19) of increase (1378% of AT8 positive cells/mm^2^ relative to the limited burden in the saline injected mice) (Figures 2AI,II). An increase in tau pathology was also detected in the cortex: 1101% in AT8 positive cells/mm^2^ (*p* = 0.01) and 102.3% AT180 positive cells/mm^2^ (trend, *p* = 0.07), relative to the limited burden in the saline injected mice (Figure 2B). Like in the tg-mice exposed to 3NP, also in the WT-mice exposed to 3NP, we detected that the increase in the pathological/phosphorylated tau in the brain was accompanied with an increase of the total tau: increase of 276.4% (*p* = 0.01) and 372.8% (*p* = 0.01) in the hippocampus and the cortex, respectively, relative to the saline injected mice (Figure 2B).

### Alterations in the Levels of GSK and Its Phosphorylation State in the Mice Exposed to 3NP

**FIGURE 3:**
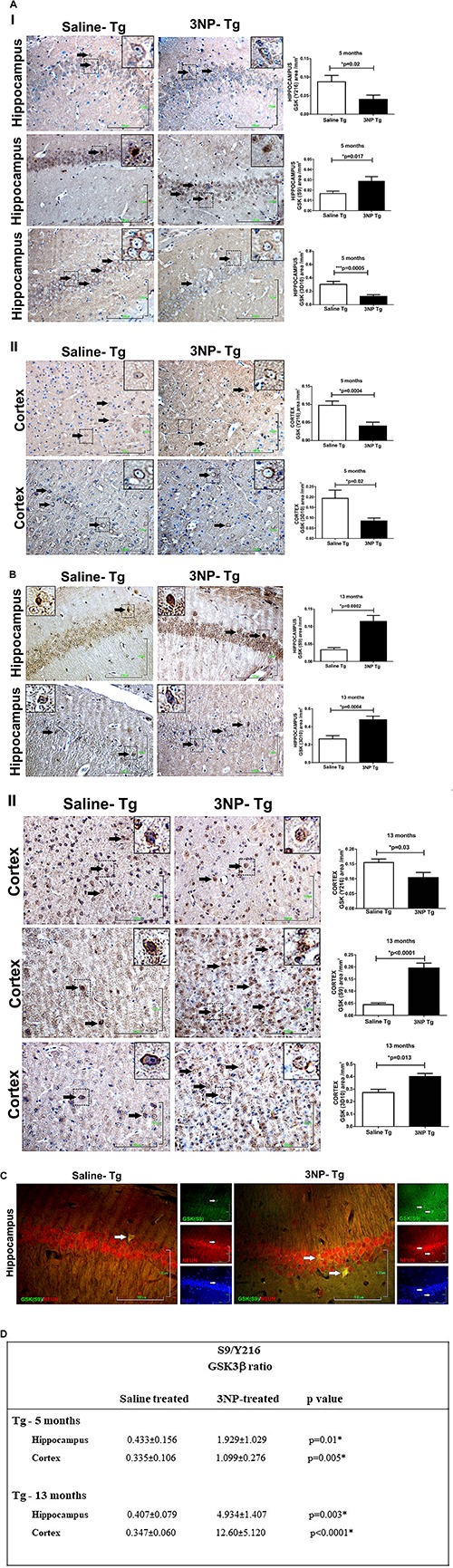
Alterations in the levels of GSK3β and its phosphorylation state in DM-Tau-tg mice exposed to 3NP. **(A)** Alterations in the brains of 5 month old 3NP-DM-Tau-tg mice under the moderate long term protocol of 3NP exposure. Significant lower GSK3β (Y216) (*p* = 0.02), higher level of GSK3β (S9) (*p* = 0.017), and lower total GSK3β (3D10 Ab, *p* = 0.0005) in the hippocampus **(I)**, with lower GSK3β (Y216) (*p* = 0.0004), and lower total GSKβ (*p* = 0.02) in the cortex **(II)**, of 3NP-DM-Tau-tg mice relative to the saline-DM-Tau-tg mice. **(B)** Alterations in the brains of 13 month old 3NP-DM-Tau-tg mice under the acute short term protocol of 3NP exposure. Significant higher level of GSK3β (S9) (*p* = 0.0002), and higher total GSK3β (*p* = 0.03) in the hippocampus **(I)**, with lower GSK3β (Y216) (*p* = 0.03), higher GSK3β (S9) (*p* < 0.0001), and higher total GSK3β (*p* = 0.013) in the cortex **(II)**, of 3NP-DM-Tau-tg mice relative to the saline-DM-Tau-tg mice. **(C)**. Collocalization of phosphorylated Ser 9 GSK3β with NeuN (neurons). Double staining of hippocampus of 13-month old 3NP-DM-Tau-tg mice exposed to the acute short term protocol of 3NP exposure for GSK3β (S9) (green), NeuN (red), and also DAPI (blue) presented. When merged, GSK3β (S9) and NeuN staining showed collocalization (yellow). Arrows show positive stained cells. Scale bars = 100 μm. Magnification of the photos in X40, dashed line indicate positive cells from each photo transferred in higher magnification insert photos. **(D)** Calculated ratio of S9/Y216 GSKβ. Increased ratio in the 3NP-treated mice relative to saline-treated mice.

We were interested to see whether the increase in tau pathology, particularly the tau phosphorylation, is regulated by the GSK3β, the key enzyme which is involved in the phosphorylation of tau characteristic to AD/tauopathies. For this purpose, we compared the levels of the GSK3β and its phosphorylated forms- which regulate its activities- in the brains of 3NP injected mice to that of saline-injected mice. Using specific Abs for total GSK3β and for the Ser 216 phosphorylated GSK3β [GSK3β (Y216)] which is associated with activated GSK3β, as well as for the Ser 9 phosphorylated GSK3β [GSK3β (S9)], associated with inactivated GSK3β – we indeed detected alterations in the level of the GSK3β and its phosphorylation states in the hippocampus and cortex of 3NP treated mice, regions in which increased tau pathology was evident in the treated mice. Interestingly, while we expected to find an increase in the GSK3β (Y216) or a decrease in GSK3β (S9) levels, other effects were noticed. Five-month old DM-Tau-tg mice exposed to the moderate long term protocol of 3NP exposure showed a significant decrease in GSK3β (Y216) (decrease of 54% area/mm^2^, *p* = 0.02, and of 58.8%, *p* = 0.0004, in the hippocampus and cortex, respectively) as well as an increase in GSK3β (S9) (increase of 75% area/mm^2^, *p* = 0.017 in the hippocampus), relative to saline injected mice, Figure 3A, with no difference in the cortex – data not shown. Ratio of S9/Y216 GSK3β was also higher in the 3NP-DM-Tau-tg mice relative to saline injected mice in the hippocampus (*p* = 0.01) and in the cortex (*p* = 0.005) (Figure 3D). This was accompanied with a decrease in the total GSK3β level (3D10) (decrease of 60.0 and 56.5% area/mm^2^ in the hippocampus and cortex, respectively, relative to saline injected mice, Figure 3A). Similar changes of decreased GSK3β (Y216) and increased GSK3β (S9) levels were detected also in the brains of the 13-month old DM-Tau-tg mice exposed to the acute short-term protocol of 3NP exposure, as follows: a decrease of 30.7% in GSK3β (Y216) area/mm^2^ in the cortex (*p* = 0.03), relative to saline treated mice, with no difference in the hippocampus – data not shown; and an increase of 336% (*p* = 0.0002), and 343.2% (*p* < 0.0001) in GSK3β (S9) area/mm^2^ in the hippocampus and cortex of 3NP-mice, respectively, relative to saline treated mice (Figure 3B). Ratio of S9/Y216 GSK3β was also higher in the 3NP-DM-Tau-tg mice relative to saline injected mice in the hippocampus (*p* = 0.003) and in the cortex (*p* < 0.0001) (Figure 3D). Double staining of GSK3β (S9) with NeuN revealed their collocalization in some of the neurons, indicating that the increase in GSK3β (S9) is taking place in the neurons (Figure 3C).

**FIGURE 4:**
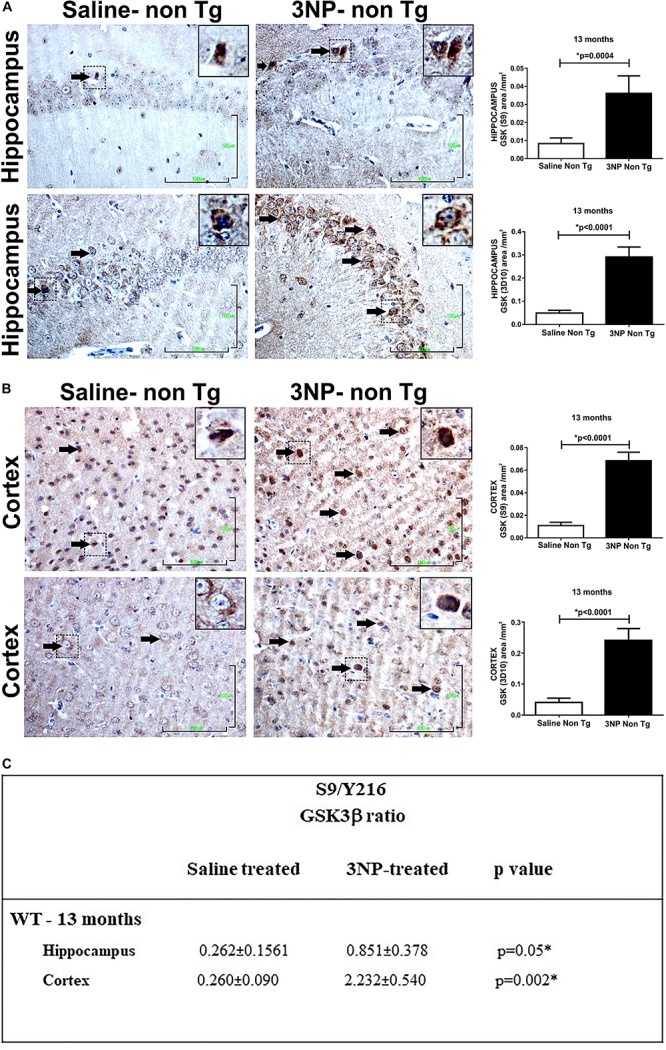
Alterations in the levels of GSK and its phosphorylation state in WT-mice exposed to 3NP. Significant higher level of GSK3β (S9) (*p* = 0.0004), and higher total GSK3β (<0.0001) in the hippocampus **(A)**, and also in the cortex **(B)**: higher level of GSK3β (S9) (<0.0001), and higher total GSK3β (<0.0001) stained cells, 3NP-WT- mice relative to saline-WT- mice. Scale bars = 100 μm. Magnification of the photos in X40, dashed line indicate positive cells from each photo transferred in higher magnification insert photos. **(C)** Calculated ratio of S9/Y216 GSKβ. Increased ratio in the 3NP-treated mice relative to saline-treated mice.

Studying the levels of the GSK3β in the 13-month old 3NP-WT-mice exposed to the acute short-term protocol of 3NP exposure, revealed similar results of increased GSK3β (S9) levels (increase of 350%, *p* = 0.004, and 580%, *p* = 0.004, in the hippocampus and cortex, respectively, relative to saline injected mice, Figure 4A), as well as increased S9/Y216 GSK3β ratio relative to saline injected mice [*p* = 0.05 and *p* = 0.002 in hippocampus and cortex, respectively (Figure 4C); with no difference in the GSK3β (Y216) – data not shown]. Taken together, these results show that in all the groups of 3NP exposure (different ages, different genotypes and 3NP-protocols) GSK3β (S9) was elevated and in some also GSK3β (Y216) was decreased (and their ratio increased). These changes in the levels of phosphorylated GSK3β forms presented by us here may fit the reports of increased GSK3β (S9) level (time dependent) under neurotoxic conditions, such as amyloid-beta and apoE4 milieu [as well as experimental diabetes, traumatic brain injury and cerebral ischemia, and some changes in GSK3β (Y216) levels (Planel et al., 2007; Zhao et al., 2012; Morales-Corraliza et al., 2016; Kisoh et al., 2017; van der Harg et al., 2017)].

We also noticed that while the 5-month old 3NP-DM-Tau-tg mice (moderate long-term protocol) showed a decrease in total GSK3β level (Figure 3A), the 13-month old 3NP-DM-Tau-tg mice (acute short-term protocol) (Figure 3B), as well as the 13-month old 3NP-WT- mice (acute short-term protocol) (Figures 4A,B) showed an increase in total GSK3β level (may be related to the different age and 3NP exposure protocols).

### Alterations in Microglial Burden and Activation State in Mice Exposed to 3NP

**FIGURE 5:**
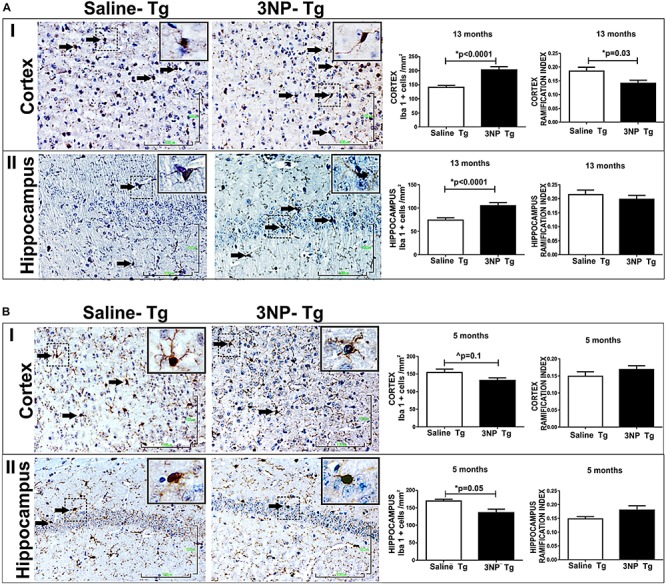
Alterations in microglial burden and activation state in DM-Tau-tg mice exposed to 3NP. **(A)** Increased microglial burden with reduced activation state in 13-month old DM-Tau-tg mice treated with an acute short-term protocol of 3NP exposure (IP injection every other for 1 month). Significant increase in burden of IbaI positive cells in the cortex of 3NP-DM-Tau-tg mice relative to saline- DM-Tau-tg mice (cells/mm^2^) (*p* < 0.0001), with a significant decreased IR of 3NP-DM-Tau-tg mice relative to saline- DM-Tau-tg mice (*p* = 0.03) **(I)**. Increase in burden of IbaI positive cells in the hippocampus of 3NP-DM-Tau-tg mice relative to saline- DM-Tau-tg mice (*p* < 0.0001), with tendency of decreased IR of 3NP-DM-Tau-tg mice relative to saline- DM-Tau-tg mice **(II)**. **(B)** Decreased microglial burden with increased activation state in 5-month old DM-Tau-tg mice treated with the moderate long-term protocol of 3NP exposure (IP injection two times a week for 5 months). Decrease in burden of IbaI positive cells in the cortex of 3NP-DM-Tau-tg mice relative to saline- DM-Tau-tg mice (trend, 0.1), with tendency of increased IR of 3NP-DM-Tau-tg mice relative to saline- DM-Tau-tg mice **(I)**. Significant decrease in burden of IbaI positive cells in the hippocampus of 3NP-DM-Tau-tg mice relative to saline- DM-Tau-tg mice (*p* = 0.005), with a tendency of increased IR 3NP-DM-Tau-tg mice relative to saline- DM-Tau-tg mice **(II)**. Arrows show positive stained cells. Scale bars = 100 μm. Magnification of the photos in X40, dashed line indicate positive cells from each photo transferred in higher magnification insert photos.

Based on the positive correlation between NFT-burden and microglial-burden in brains of tauopathy patients (Ishizawa and Dickson, 2001) and as reported by us in NFT-Tg mice (Boimel et al., 2009), as well as the finding that microglial activation precede tangles in a tauopathy mouse model (Yoshiyama et al., 2007), we were interested to see whether the increase of tau pathology following the exposure to 3NP of DM-Tau-tg mice as well as the generation *de novo* of NFTs in the non-tg mice after 3NP exposure is associated with a microglial response. Such an association is relevant particularly since 3NP has been shown to affect microglial burden in Huntington’s disease (HD) (Chakraborty et al., 2014). Using the Iba1 staining for microglia we detected an increase in microglial burden [increase of 43.8% (*p* < 0.0001) and 41.87% (*p* < 0.0001) in the cortex and the hippocampus, respectively, relative to the saline treated mice], and a decrease in the activation state (RI) [a decrease of 23.8% (*p* = 0.03) and 8%, respectively] in the 13-month old 3NP DM-Tau-tg mice (under the acute short term protocol of 3NP treatment) (Figure 5A). Analyzing the M1/M2 phenotype of the microglia revealed no specific response of iNos/Arg1 (data not shown).

**FIGURE 6:**
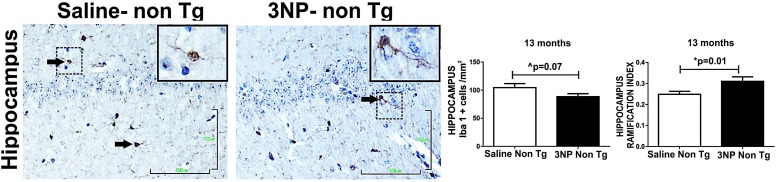
Alterations in microglial burden and activation state in WT-mice exposed to 3NP. Decreased burden of IbaI positive cells in the hippocampus of 3NP-WT-mice relative to saline- WT- mice (cells/mm^2^) (trend *p* = 0.07), with significant increased IR of 3NP-WT- mice relative to saline- WT- mice (*p* = 0.01). Arrows show positive stained cells. Scale bars = 100 μm. Magnification of the photos in X40, dashed line indicate positive cells from each photo transferred in higher magnification insert photos.

The effect of 3NP on microglia was different in the 5-month old 3NP DM-Tau-tg mice (under the moderate long- term protocol of 3NP treatment). A significant decrease in microglial burden was detected [decrease of 19.4% (*p* = 0.005) and 14.6% (trend, *p* = 0.1) in the hippocampus and cortex, respectively, relative to the saline treated mice, with tendencies of increased activation state [increase of 1117 and 13.5% in the hippocampus and in the cortex, respectively] (Figure 5B). Similar effects were noticed when testing the microglial response in the 13-month old mice 3NP-WT mice (under the acute short-term protocol of 3NP treatment): an increase in RI of 21.4% (*p* = 0.01), with a decrease of 15.6% (trend *p* = 0.07) in cell burden/mm^2^ in the hippocampus, relative to the saline treated mice (Figure 6). No specific M1/M2 phenotype was detected (data not shown), as it was the case in the 3NP- DM-Tau-tg mice.

Taken together, these results point to the responsiveness of the microglia to 3NP exposure (an exposure which increased the tau-pathology), by a response varying from increase in microglial burden with reduced activation state to a response of decreased burden with increased activation state.

## Discussion

We set up to test in tangle- tg mice the effect of oxidative stress induced by the 3NP mitochondrial inhibitor, a toxin which has been reported to induce also microglial activation in the striatum in HD (Chakraborty et al., 2014) and MSA (Stefanova et al., 2005) mouse models, and in rats (Ryu et al., 2003). As such, this toxin mimics mitochondrial dysfunction, oxidative stress, and microglial activation, processes which are taking place in AD and tauopathy, therefore, making it a preferential toxin for studying the tangle pathogenesis.

We used the tangle-tg mice (DM-Tau tg mice), previously generated by us (Rosenmann et al., 2008), with the advantage of studying the effect of the toxin under physiological and tolerated expression levels of mutant tau tg (due to its regulation by the original tau promoter), and not under overexpression levels of a tau-Tg. We used two different protocols (acute and moderate) of 3NP exposure, for addressing the effect of the 3NP toxin in different ages at onset, different intensities and duration of treatments. We also used WT mice to address whether the 3NP affects also non-mutant tau. Our results show that 3NP exposure increased the tangle pathology in cortex and hippocampus of the DM-Tau tg mice, in the different protocols tested, as expressed by Gallyas- or phosphorylated-tau positive cell increased burden. This points that the responsiveness of accelerating tau pathology under the 3NP treatment is quite a steady one, taking place under different conditions: short term or long term delivery, low or high frequency of delivery, when starting treatment before spontaneously developing mutant tau abnormality or when is already highly developed in aged mice. This suggests a high vulnerability of hippocampal and cortical neurons to mitochondrial inhibition/oxidative stress caused by this toxin, to develop tangle-pathology. Moreover, this was not limited only to mutant human tau, which is predisposed to become phosphorylated and aggregate as tangles in the tau Tg mice (3NP DM-Tau tg mice), but also WT-mice, which express only endogenous non-mutant mouse tau, developed tangle-pathology under exposure to 3NP. This *de novo* generation of tau pathology in WT-mice, which otherwise are free of neurodegenerative pathology, shows the strong impact of mitochondrial dysfunction/oxidative stress on tangle-formation.

Our study showing that the mitochondria toxin induces tau pathology is in accord with the study in rats showing an increase in phosphorylated tau (and α-synuclein) in striatum after exposure to another mitochondrial inhibitor, the complex I inhibitor rotenone (Höglinger et al., 2005). This may shed some light on the controversies regarding the different responses of tau pathology to various oxidative stress stimuli (Ko et al., 1997; Mattson et al., 1997; Olivieri et al., 2000, 2001; LoPresti and Konat, 2001; Egaña et al., 2003; Gómez-Ramos et al., 2003; Ho et al., 2003; Galas et al., 2006; Poppek et al., 2006; Dias-Santagata et al., 2007; Melov et al., 2007; Ohsawa et al., 2008; Benítez-King et al., 2010; Kulic et al., 2011: Taga et al., 2011; Yoshiike et al., 2012), pointing that oxidative stress stimuli in the form of mitochondrial inhibition do enhance tau pathology. This notion may gain support also from the studies in genetic mouse models defective in mitochondria enzymes or factors, showing increased phosphorylated tau burden (Melov et al., 2007; Ohsawa et al., 2008; Kulic et al., 2011).

That mitochondrial dysfunction is associated with the tau pathology is well established (reviewed in García-Escudero et al., 2013), presenting dual interaction in which the presence of pathological tau induced mitochondrial deficits such as inhibition of trafficking and shape elongation leading to cell death (Ebneth et al., 1998; Stamer et al., 2002; DuBoff et al., 2012), and down regulation of proteins involved in mitochondrial respiration and metabolism, antioxidant enzymes, and increased ROS production (David et al., 2005; Dumont et al., 2011) in cells and in animal models; yet these changes preceded tau hyperphosphorylation and tangle formation. Our results showing that external exposure to the mitochondrial inhibitor increases tau pathology in DM-Tau-tg mice and WT-mice presents actually the other direction of the tau-mitochondrial dysfunction dialogue, in which phosphorylation of tau follows the mitochondrial dysfunction. This may be in accord with the report that mitochondrial dysfunction in microglial cells contributed to the detrimental effects seen in neurodegenerative diseases (Ferger et al., 2010).

The increase in tangle-pathology under exposure to 3NP was accompanied by an increase in microglial burden or its activation state, presenting a complex and actually a moderate response of either increase in microglial burden together with decrease in activation state, or decrease in microglial burden associated with increase in activation state. The difference in the mode of response of microglia and its extent between the different groups exposed to the 3NP, may be attributed to the different ages and treatment protocols as well as mouse strains. That microglial functions differ between DM-Tau-tg mice and WT-mice has been reported by us previously (Lahiani-Cohen et al., 2011). That microglial response is a complex phenomenon with a wide spectrum of responses is becoming more and more clear, as also reported in studies showing that 3NP can trigger both activation and also apoptotic cell death of microglia in adult rats (Ryu et al., 2003), as well as studies showing age dependent and brain region specific response of the microglial cells (Ritzel et al., 2015). Yet, it is well known that microglia may lead to an increase in secretion of inflammatory cytokines, which can induce phosphorylation of tau (Li et al., 2003; Quintanilla et al., 2004; Bhaskar et al., 2010), or by alteration in secretion of growth factors like brain derived neurotrophic factor (BDNF) which affects tau phosphorylation (via regulation of GSK3β) (Mai et al., 2002; Elliott et al., 2005; Lahiani-Cohen et al., 2011). Moreover, activated microglia can contribute to the spread of pathological tau (Maphis et al., 2015). Other studies showed other aspects of the dialogue between tau and microglia. Microglial activation has been reported to precede tangle formation in P301S tau tg mice (Yoshiyama et al., 2007; Dumont et al., 2011), and that tau itself can activate microglia (Wang et al., 2013). These studies, altogether, point to a dual dialogue between microglia and tau protein/pathology.

Trying to see whether the increase in tau-pathology under 3NP involves the regulation of GSK, the key kinase which is involved in the phosphorylation of tau in AD/tauopathy related epitopes, we indeed detected alterations in the enzyme level and its phosphorylated state. While one might expect a decrease in the GSK3β (S9) (considered to inactivate the kinase activity) with an increase in the Ser 216 phosphorylated GSK3β isoform (considered to activate the kinase activity) to accompany the increased tau phosphorylation under 3NP exposure, we detected an increase in the GSK3β (S9) in the 3NP treated mice with each of the protocols (acute and moderate), with the 3NP-DM- Tau-tg-mice showing also a decrease in GSK3β (Y216). This seems to be in accord with the similar regulation taking place under neurotoxic conditions, such as under exposure to amyloid-beta or apoE4 (Cedazo-Mínguez et al., 2003), as well as in experimental diabetes, traumatic brain injury, and cerebral ischemia in rodents (Planel et al., 2007; Zhao et al., 2012; Morales-Corraliza et al., 2016; Kisoh et al., 2017; van der Harg et al., 2017), where at early stages following model induction indeed the expected decrease in GSK3β (S9) [with also an increase in GSK3β (Y216) was detected, but at latter stages the opposite effects were detected]. These time dependent effects may point to an early stage of damage taking place and further followed by attempts for correction. This phenomenon was described as “the unbalanced regulation of which may contribute to AD pathology” (Cedazo-Mínguez et al., 2003). These alterations in GSK3β detected by us may suggest that while at earlier stages a decrease in GSK3β (S9)/increase in GSK3β (Y216) might have taken place and induced tau phosphorylation, at latter stages (after 1 or 5 months of exposure) there was some possible correction in the GSK3β regulation. Additional studies using inhibitors of GSK3β will allow to clarify the involvement of GSK3β in the 3NP-induced tau phosphorylation. Yet, it is possible that other mechanisms are involved in the increased tau phosphorylation/pathology under exposure to 3NP.

It seems that the increase in pathological/phosphorylated tau in response to exposure to 3NP was accompanied with an increase in total tau (particularly in the 13-month old- mice in cortex of tg-mice, while in both hippocampus and cortex of WT-mice, yet only some trend, if at all, in the hippocampus of 5-month old Tg-mice). This may point to a possible effect of 3NP on the accumulation of total tau (either increased expression or reduced degradation), an effect which may be age related. It can also be speculated that a decrease in the functional tau (due to its increased phosphorylation) under exposure to 3NP led to the increase in the expression of tau protein.

Our study shows the effect of increased tau-pathology by exposure to 3NP by using different approaches: studying tauopathy tg-mice and WT-mice, using different protocols of exposure (age, duration, and frequency), and performing various histological and immunohistochemical analysis. Adding other methods may provide further support. In order to pinpoint whether the different effects of 3NP on the GSK3β and microglia is driven by the difference of age or duration, etc – additional studies are needed, such as exposing WT-mice to 3NP at a younger age.

Exposing the DM-Tau tg mice to 3NP provides not only a tool to study the effect of mitochondrial dysfunction/oxidative stress on tangles, but can provide a tangle model for genetic/environmental interaction by using genetic predisposition and environmental exposure, thereby representing more complex forms of the disease (getting closer to sporadic form), which are not pure genetic.

Although oxidative stress is implicated in the development of tau pathology and neurodegeneration, many anti-oxidant agents have been studied however so far without significant clinical success (Frank and Gupta, 2005; Forbes et al., 2015). Also targeting microglia, using the microglial inhibitor minocycline, revealed controversial results (Noble et al., 2009; Parachikova et al., 2010; Cai et al., 2013; Möller et al., 2016). It is possible that mitochondrial dysfunction is involved in the interplay of the tau pathology with oxidative stress as well as with microglial response, and as such - a mitochondrial therapy should be considered as a potential target in this regard. Our results showing the harmful effect of the mitochondrial inhibitor 3NP on tangle pathology supports this notion. This may be relevant to the diseases of tau pathology, not only AD but also to the primary tauopathies (FTDP, Pick etc.).

## Data Availability Statement

The datasets generated for this study are available on request to the corresponding author.

## Ethics Statement

The experiments were approved by the Institutional Ethics Committee of The Hebrew University of Jerusalem.

## Author Contributions

HR designed and conceived the experiments, and wrote the manuscript. IL-C performed the experiments. NG, OT, and RL conducted the histological studies. All authors read and approved the manuscript.

## Conflict of Interest

The authors declare that the research was conducted in the absence of any commercial or financial relationships that could be construed as a potential conflict of interest.
