# Supplementary material for: Exposure to 3-Nitropropionic Acid Mitochondrial Toxin Induces Tau Pathology in Tangle-Mouse Model and in Wild Type-Mice
Source: Front Cell Dev Biol. 2020 Jan 14;7:321. doi: 10.3389/fcell.2019.00321 (PMC6971403; doi:10.3389/fcell.2019.00321)
Supplement: Supplementary file 1 [file Table_1.DOCX]

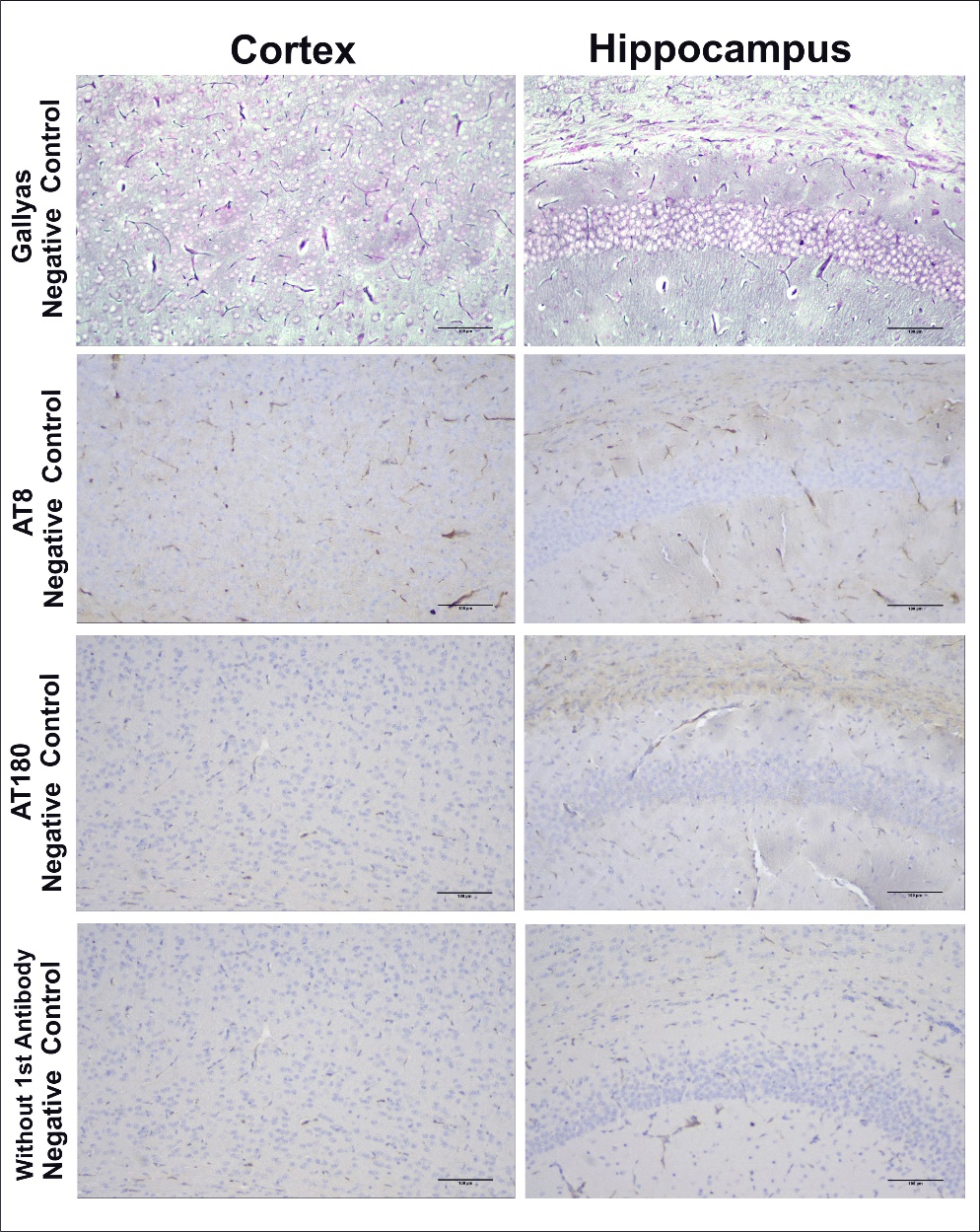


**Supplement fig. 1: Negative control staining for Gallyas, AT8 and AT180**. No tangle or phos-tau pathology was detected in the brain tissue of 17 days old naïve WT-mice (some signal appeared in the blood vessels, as the mice were sacrificed without transacardial perfusion). Staining without 1st Ab showed no signal.
